# Effects of nutritional supplementation on physical performance and sport-specific skills in volleyball players: a systematic review and meta-analysis

**DOI:** 10.3389/fphys.2026.1763606

**Published:** 2026-03-05

**Authors:** Bingran Zhao, Haiting Zhai

**Affiliations:** 1 Sports Coaching College, Beijing Sports University, Beijing, China; 2 Naval Aviation University, Yantai, China

**Keywords:** meta-analysis, nutritional supplementation, physical performance, sports performance, sport-specific skills, volleyball

## Abstract

**Background:**

Nutritional supplementation is widely used to support sports performance; however, evidence specific to volleyball players remains fragmented. This systematic review and meta-analysis aimed to evaluate the effects of nutritional supplementation on physical performance and sport-specific skills in competitive volleyball players.

**Methods:**

Following PRISMA guidelines, five electronic databases were systematically searched. Randomized controlled trials investigating acute or chronic nutritional supplementation in healthy volleyball players were included. Outcomes included vertical jump performance, muscle strength, agility, and sport-specific technical skills. Risk of bias was assessed using the Cochrane tool, and random-effects meta-analyses were performed.

**Results:**

Thirteen randomized controlled trials involving 240 participants were included. Nutritional supplementation was associated with significant improvements in vertical jump performance (SMD = 0.47, 95% CI: 0.24–0.70), muscle strength (SMD = 0.43, 95% CI: 0.17–0.69), agility (SMD = 0.89, 95% CI: 0.54–1.24), and sport-specific technical skills (SMD = 0.63, 95% CI: 0.31–0.95). Subgroup analyses indicated beneficial effects following both acute and chronic supplementation protocols, with minimal statistical heterogeneity across outcomes.

**Conclusion:**

Within the available evidence, nutritional supplementation may contribute to improvements in selected physical performance and sport-specific skill outcomes in competitive volleyball players. These findings may inform evidence-based nutritional strategies aimed at supporting sports performance in volleyball, while highlighting the need for larger, ecologically valid trials.

## 1 Introduction

Volleyball is a high-intensity, intermittent team sport characterized by frequent explosive actions such as jumping, spiking, blocking, and rapid changes in direction (Sheppard et al., 2007; Silva et al., 2014). In contrast to continuous endurance sports, elite volleyball matches typically last between 60 and 150 min, during which athletes perform 60 to 100 maximal jumps and numerous high-velocity defensive movements, all while maintaining cognitive vigilance for tactical decision-making (Mroczek et al., 2017; Sattler et al., 2015). The sport’s physiological demands require a unique combination of anaerobic power for execution and aerobic capacity for recovery between rallies (Langan-Evans et al., 2011). With the increasing intensity of competitive schedules and the narrowing margins of victory, athletes and practitioners are increasingly seeking effective ergogenic strategies to optimize performance, delay fatigue, and accelerate recovery (Bishop, 2010; Hopkins et al., 1999).

Nutritional supplementation is widely used to enhance training adaptations and match performance (Peeling et al., 2018). The efficacy of specific supplements, such as caffeine, creatine, and β-alanine, has been recognized by organizations like the International Olympic Committee and International Society of Sports Nutrition (Kerksick et al., 2018; Maughan et al., 2018). Caffeine enhances central nervous system drive and neuromuscular recruitment (Davis and Green, 2009), while creatine and β-alanine improve muscle energy availability and buffering capacity, respectively (Kreider et al., 2017; Trexler et al., 2015). These mechanisms align with volleyball’s high-intensity demands, where the phosphagen system supports explosive power and glycolysis aids sustained efforts (Zawieja et al., 2023).

Despite a growing body of research, the application of nutritional interventions to volleyball remains limited. Most systematic reviews and meta-analyses have focused on endurance sports (e.g., cycling, running) or continuous high-intensity team sports like soccer and rugby (Grgic, 2022; Wang et al., 2023). Research on volleyball-specific nutritional effects is scarce and often limited to gross motor outputs, such as jump height, neglecting crucial aspects like agility, on-court movement speed, and technical skills (Gabbett and Georgieff, 2007; Del Coso et al., 2014). While studies have shown benefits of supplements like caffeine for agility (Kaszuba et al., 2023) and β-alanine for repeated jumps (Guo and Wang, 2024), there is still no consensus on which supplement—acute neuro-stimulants or chronic metabolic conditioners—offers the most significant benefits for volleyball’s unique intermittent demands (Nemati et al., 2023).

The primary aim of this systematic review and meta-analysis was to evaluate the efficacy of nutritional supplementation on physical performance outcomes, including vertical jump, strength, agility, and sport-specific technical skills, in competitive volleyball players. A secondary aim was to explore, through subgroup analyses, whether supplementation protocol (acute vs. chronic) and supplement type influence the magnitude of these effects. Ultimately, the findings aim to provide evidence-based recommendations for optimizing nutritional strategies for volleyball athletes.

## 2 Materials and methods

This systematic review and meta-analysis followed the PRISMA guidelines (Page et al., 2021) and was prospectively registered with PROSPERO (registration number CRD420251177356).

### 2.1 Eligibility criteria

This systematic review adhered to the PRISMA guidelines, with the PRISMA 2020 Checklist provided in Supplementary Material S1. The eligibility criteria were defined based on the PICOS framework.

Inclusion criteria: (I) Studies involving healthy male or female volleyball players. (II) Studies evaluating nutritional supplementation strategies, such as protein, creatine, caffeine, beta-alanine, branched-chain amino acids, nitrate, vitamin D, or multi-vitamin/mineral complexes, with quantified intake. Both acute and chronic supplementation protocols administered alongside volleyball-specific or physical training were included. (III) Acute and chronic supplementation trials, provided that dosage was quantified and a placebo/control condition under identical training/testing procedures was used. (IV)Studies with a control group receiving a placebo, normal diet, or no supplementation, under identical training conditions. (V) Primary outcomes focusing on objective measures of physical performance and sport-specific skills, such as jump performance, agility and strength (e.g., one-repetition maximum in bench press or squat).

Exclusion criteria: (I) Studies involving children (<16 years), pregnant women, elderly individuals (>65 years), or individuals with clinical conditions; (II) Studies that included major combined interventions that prevented isolating the supplement’s effect; (III) Studies that only reported subjective measures without objective performance metrics; (IV) Studies published as abstracts, conference proceedings, theses, editorials, method papers, or duplicate publications.

### 2.2 Information sources

Comprehensive literature searches were conducted across five major electronic databases: Web of Science, PubMed, Embase, The Cochrane Library, and Scopus. To ensure the inclusion of all relevant studies, additional manual searches were performed on reference lists of included articles and relevant reviews. The final search update was completed on 8 December 2025. No restrictions regarding the publication date were applied during the search process.

### 2.3 Search strategy

The search strategy employed a combination of Medical Subject Headings and free-text keywords related to volleyball, nutritional supplementation, and athletic performance variables. Boolean operators were utilized to combine search terms effectively, and the specific search string developed for PubMed was adapted for use in the other databases to account for syntax differences. The detailed search strings and combinations for each database are provided in Supplementary Table S2.

### 2.4 Study selection

The literature search and screening process were independently conducted by two authors (B.R. and H.T.). Any discrepancies regarding study selection were resolved through discussion or consultation with a third reviewer (Y.H.) to reach a consensus.

### 2.5 Data collection process

Data extraction was performed independently by one author (B.R.) and subsequently verified by a second author (H.T.). Any inconsistencies were adjudicated through consultation with a third reviewer (Y.H.). Extracted data included bibliographic details, participant characteristics, study design, intervention protocols, and placebo specifications. Quantitative synthesis focused on the following specific outcomes: vertical jump height, muscle strength, agility, and sport-specific technical skills. Sport-specific technical skills were operationalized as objective ball-related performance outcomes, including spike-ball velocity and accuracy-based skill-test scores. In cases where numerical data were not reported in the text or tables, the corresponding authors were contacted. If raw data remained unavailable, values were extracted from graphical representations using WebPlotDigitizer software (version 4.6; Ankit Rohatgi, Pacifica, CA, United States) (Burda et al., 2017).

### 2.6 Data synthesis and unit-of-analysis considerations

To minimize unit-of-analysis errors, each study contributed at most one effect size per outcome domain (vertical jump, strength, agility, and sport-specific technical skills) in the primary analyses. For cross-over trials, within-participant effect sizes were calculated when paired data were available; otherwise, trials were conservatively analyzed as parallel-group studies by treating intervention and placebo conditions as independent groups. The robustness of this approach was examined through sensitivity analyses.

For multi-arm trials, control groups were split or intervention arms were combined in accordance with Cochrane recommendations to avoid double-counting participants. When studies reported multiple supplementation doses, dose arms were combined into a single comparison for the primary analysis. If combination was not feasible, a single dose was selected based on predefined clinical relevance and common usage rather than effect magnitude, and alternative dose selections were explored in sensitivity analyses. These procedures were prespecified to reduce selective outcome reporting and to preserve the independence of effect sizes.

Given the diversity of supplementation strategies, the primary analyses summarized the overall ‘supplementation vs. control’ effect within each outcome domain using a random-effects model, while subgroup analyses were prespecified by protocol timing (acute vs. chronic) and supplement class to aid interpretability. Subgroup findings were considered exploratory when based on a small number of studies.

### 2.7 Quality assessment

The methodological quality and risk of bias for the included randomized controlled trials were assessed by two independent reviewers (B.R. and H.T.) using the Cochrane Collaboration’s tool for assessing risk of bias (Higgins et al., 2011). This tool evaluates bias across seven specific domains: random sequence generation, allocation concealment, blinding of participants and personnel, blinding of outcome assessment, incomplete outcome data, selective reporting, and other sources of bias. Each domain was judged as having a “low,” “high,” or “unclear” risk of bias. Disagreements in the risk grading were resolved through consensus or by involving a third investigator (Y.H.). Additionally, potential publication bias was visually inspected using funnel plots.

### 2.8 Meta-analysis calculations

Meta-analytic calculations and forest plot generation were performed using Review Manager (RevMan) software (Version 5.4, The Cochrane Collaboration, 2020). Since the included studies utilized varying measurement scales for physical performance, the standardized mean difference (SMD) with 95% confidence intervals (CI) was calculated using a random-effects model (DerSimonian and Laird, 1986). Effect sizes were interpreted as trivial (<0.2), small (0.2–0.5), moderate (0.5–0.8), or large (>0.8) (Cohen, 1988). Statistical heterogeneity among studies was assessed using the I^2^ statistic, where values of 25%, 50%, and 75% represented low, moderate, and high heterogeneity, respectively (Higgins et al., 2003). Subgroup analyses were conducted based on intervention duration and supplement type. Statistical significance was set at p < 0.05.

## 3 Results

### 3.1 Study selection

**FIGURE 1:**
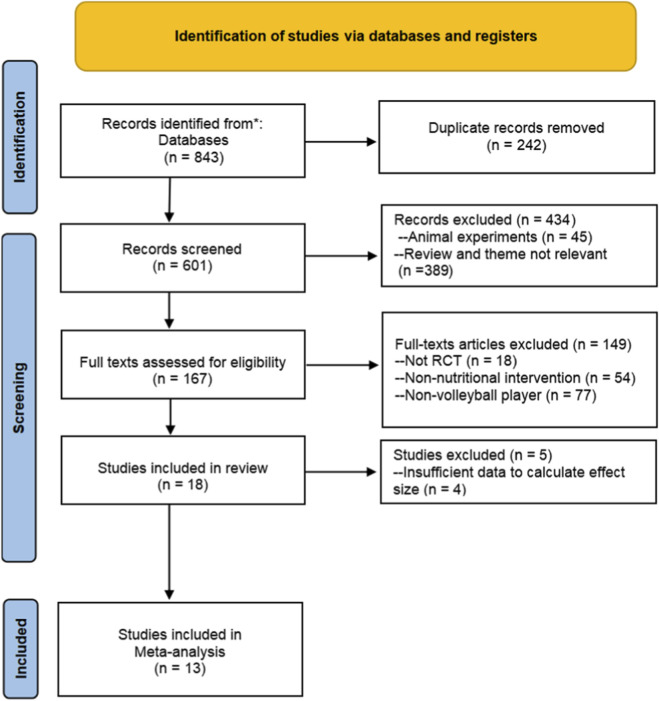
Flowchart of search strategy and article selection process.

Our search across five databases yielded 843 records. After removing 242 duplicates, 601 studies were screened. Based on titles and abstracts, 434 records were excluded. Consequently, 167 full-text articles were assessed, and 149 were excluded for not meeting eligibility criteria: non-Randomized Controlled Trials design (n = 18), non-nutritional intervention (n = 54), or non-volleyball participants (n = 77). 18 studies met the inclusion criteria and were included in the qualitative synthesis. Of these, 13 studies provided sufficient outcome data to calculate effect sizes and were therefore included in the meta-analysis (Del Coso et al., 2014; Guo and Wang, 2024; Kaszuba et al., 2023; Lamontagne-Lacasse et al., 2011; Martín-Martínez et al., 2020; Martins et al., 2020; Mielgo-Ayuso et al., 2015; Nemati et al., 2023; Pérez-López et al., 2015; Portal et al., 2011; Setaro et al., 2014; Wang et al., 2025; Zbinden-Foncea et al., 2018). The remaining five studies were not quantitatively synthesized because effect sizes could not be calculated due to insufficient reporting of outcome data (e.g., missing dispersion statistics and non-extractable results). The selection process is shown in Figure 1.

### 3.2 Participants and study general characteristics

The included studies spanned from 2011 to 2025. Six studies were published from 2011 to 2015. The remaining seven studies were published from 2018 to 2025. All 13 studies were randomized clinical trials. Most included studies were described as placebo-controlled, and several reported double-blinding procedures; however, blinding integrity could not be verified consistently due to incomplete reporting. Six studies utilized a cross-over design, while the remaining seven employed parallel groups. The sample sizes varied, ranging from 8 to 48 participants, resulting in a total of 240 participants with a median of 15. Among these participants, 78% were males (n = 188), whereas 22% were females (n = 52). The remaining nine studies involved only male participants. Regarding the training status of participants, all studies involved elite, professional, or collegiate-level volleyball players engaged in regular training and competition. The mean age of the overall sample was 21.0 years. The general characteristics of each study can be found in Table 1.

**TABLE 1:** Characteristics of the included studies.

Study (first author, year)	Sample size (Exp/Con)	Age (years)	Body mass (kg)	Supplement type	Intervention protocol	Duration	Outcome measures
Del Coso et al. (2014)	15/15 (cross-over)	21.8 ± 6.9	79.6 ± 11.0	Caffeine	3 mg/kg	Acute (60 min pre)	VJ, strength, agility, Game Perf.
Guo and Wang (2024)	10/10	24.6 ± 2.5	81.5 ± 4.1	β-Alanine	4.8 g/day	8 weeks	VJ, sprint, agility, VO2max
Kaszuba et al. (2023)	12/12 (cross-over)	23 ± 3	85.9 ± 11.2	Caffeine	3.2 ± 0.4 mg/kg (gum)	Acute (15 min pre)	VJ, sprint, agility, skill accuracy
Lamontagne-Lacasse et al. (2011)	4/4	22 ± 1.5	84 ± 8	Creatine	20 g/d (4 days) + 5 g/d (24 days)	4 weeks	VJ (block jump endurance)
Martín-Martínez et al. (2020)	6/6	23.8 ± 2.2	84.5 ± 15.1	BCAA	21 g/week (7 g/day, 3 days/wk)	1 week	VJ (CMJ)
Martins et al. (2020)	12/12 (cross-over)	16.5 ± 0.6	77.5 ± 8.4	Grape juice	400 mL/day	14 days	Power, oxidative stress
Mielgo-Ayuso et al. (2015)	11/11	27.0 ± 5.6	70.2 ± 7.9	Iron	325 mg/day (ferrous sulphate)	11 weeks	Strength, hematology
Nemati et al. (2023)	15/15/15 (cross-over)	20.8 ± 1.0	70.2 ± 6.9	Caffeine	3 mg/kg and 6 mg/kg	Acute (60 min pre)	VJ, strength, agility, skill score
Pérez-López et al. (2015)	13/13 (cross-over)	25.2 ± 4.8	64.4 ± 7.6	Caffeine	3 mg/kg	Acute (60 min pre)	VJ, strength, agility, Game Perf.
Portal et al. (2011)	14/14	16.1 ± 1.3	72.3 ± 10.3	HMB	3 g/day	7 weeks	VJ, strength, Body Comp.
Setaro et al. (2014)	12/13	17.4 ± 1.2	83.0 ± 9.5	Magnesium	350 mg/day	4 weeks	VJ, lactate
Wang et al. (2025)	12/12/12/12	20.0 ± 1.0	78.0 ± 5.0	RHO + CAF	RHO: 2.4 g/d; CAF: 3 mg/kg	4 weeks (RHO); acute (CAF)	VJ, RPE
Zbinden-Foncea et al. (2018)	10/10 (cross-over)	18.8 ± 2.0	85.2 ± 10.1	Caffeine	5 mg/kg	Acute (60 min pre)	VJ (CMJ metrics)

Exp, Experimental group; Con, Control group; NR, not reported; VJ, vertical jump; CMJ, countermovement jump; BCAA, Branched-chain amino acids; HMB, β-hydroxy-β-methylbutyrate; RHO, rhodiola rosea; CAF, caffeine; RPE, rating of perceived exertion; Game Perf., Game Performance. Data are presented as Mean ± SD., For multi-arm or multi-dose trials, data handling for quantitative synthesis followed the predefined unit-of-analysis approach described in Section 2.6 to avoid double-counting participants.

### 3.3 Intervention characteristics

Intervention characteristics can be found in Table 1. Five studies solely focused on acute caffeine supplementation administered via energy drinks or capsules. One study evaluated caffeine delivery via chewing gum (Kaszuba et al., 2023). Single studies assessed the effects of Beta-alanine (Guo and Wang, 2024), Creatine (Lamontagne-Lacasse et al., 2011), Magnesium (Setaro et al., 2014), Iron (Mielgo-Ayuso et al., 2015), HMB (Portal et al., 2011), BCAA (Martín-Martínez et al., 2020), and Grape Juice (Martins et al., 2020). One study assessed the effects of Caffeine, Rhodiola rosea, and combined Caffeine + Rhodiola supplementation (Wang et al., 2025). For analytical consistency, the caffeine-only condition in Wang et al. (2025) was treated as an acute intervention, whereas the Rhodiola-only and combined conditions were treated as chronic interventions. These categorizations were applied at the subgroup level, and the unit-of-analysis approach described in Section 2.6 was used to avoid double-counting the control condition. Similarly, in the study by Nemati et al. (2023), both 3 mg/kg and 6 mg/kg caffeine doses were reported. For the primary meta-analysis, dose arms were combined into a single caffeine comparison to avoid selective inclusion; where applicable, sensitivity analyses were conducted using a predefined single-dose selection to assess robustness.

Caffeine doses ranged from 3.0 to 6.0 mg/kg, with the most frequently used dose being 3.0 mg/kg. Supplements were administered in various forms: dissolved in sports drinks or water, encapsulated, or as chewing gum. Placebo was administered in matching forms: either as a non-caffeinated beverage, mixed with water, or as inactive placebo capsules containing substances like dextrose, polydextrose, or flour. The duration of the interventions varied significantly, ranging from acute single-dose administration (typically 60 min pre-exercise) to chronic supplementation lasting up to 11 weeks (Mielgo-Ayuso et al., 2015).

### 3.4 Quality assessment

**FIGURE 2:**
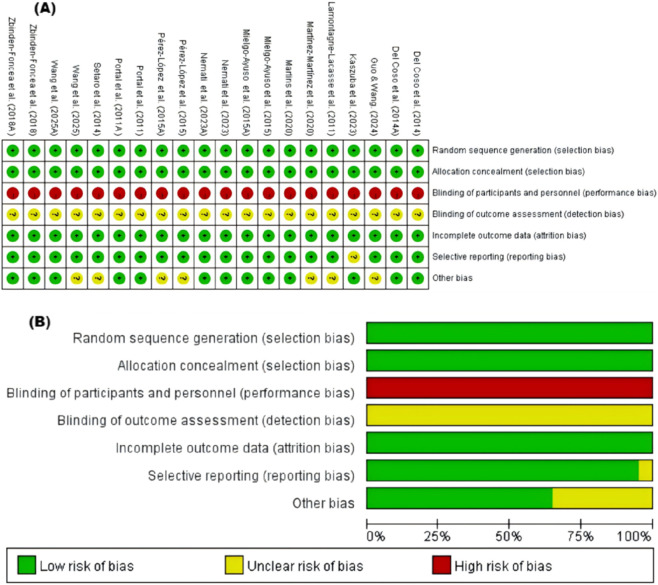
Methodological quality. **(A)** traffic-light plot of individual studies **(B)** domain-level summary.

The risk of bias assessment is presented in Figures 2A,B. All included studies were judged as having a low risk of selection bias for random sequence generation and allocation concealment, and attrition bias was also rated as low across trials. Most studies were classified as low risk for selective reporting. In contrast, performance bias was rated as high because participant blinding is difficult to maintain in supplementation trials when interventions produce perceptible physiological effects, which can influence expectations and effort during performance testing. Detection bias was generally judged as unclear because blinding of outcome assessors was insufficiently described, preventing a confident judgment.

### 3.5 Performance tests

Included studies utilized a variety of exercise performance tests. Vertical jump performance was measured in 11 studies (Del Coso et al., 2014; Guo and Wang, 2024; Kaszuba et al., 2023; Martín-Martínez et al., 2020; Martins et al., 2020; Nemati et al., 2023; Pérez-López et al., 2015; Portal et al., 2011; Setaro et al., 2014; Wang et al., 2025; Zbinden-Foncea et al., 2018). Regarding muscle strength, handgrip strength was measured in four studies (Del Coso et al., 2014; Martins et al., 2020; Nemati et al., 2023; Pérez-López et al., 2015); isokinetic joint torque in two studies (Portal et al., 2011; Setaro et al., 2014); and one-repetition maximum or repetition maximum (RM) tests in two studies (Mielgo-Ayuso et al., 2015; Portal et al., 2011). Agility were evaluated using the T-test (standard or modified) in four studies (Del Coso et al., 2014; Guo and Wang, 2024; Kaszuba et al., 2023; Pérez-López et al., 2015). Sport-specific technical skills were assessed via accuracy scoring for serving or spiking in two studies (Kaszuba et al., 2023; Nemati et al., 2023) and simulated game performance analysis in two studies (Del Coso et al., 2014; Pérez-López et al., 2015). When multiple strength (or jump/agility) measures were reported within the same study, a predefined hierarchy was applied to select one representative measure per domain for the primary analysis, with alternative measures examined in sensitivity analyses. Several outcomes, particularly agility and technical skills, were predominantly informed by acute caffeine trials, whereas non-caffeine supplements were sparsely represented.

#### 3.5.1 Vertical jump performance

**FIGURE 3:**
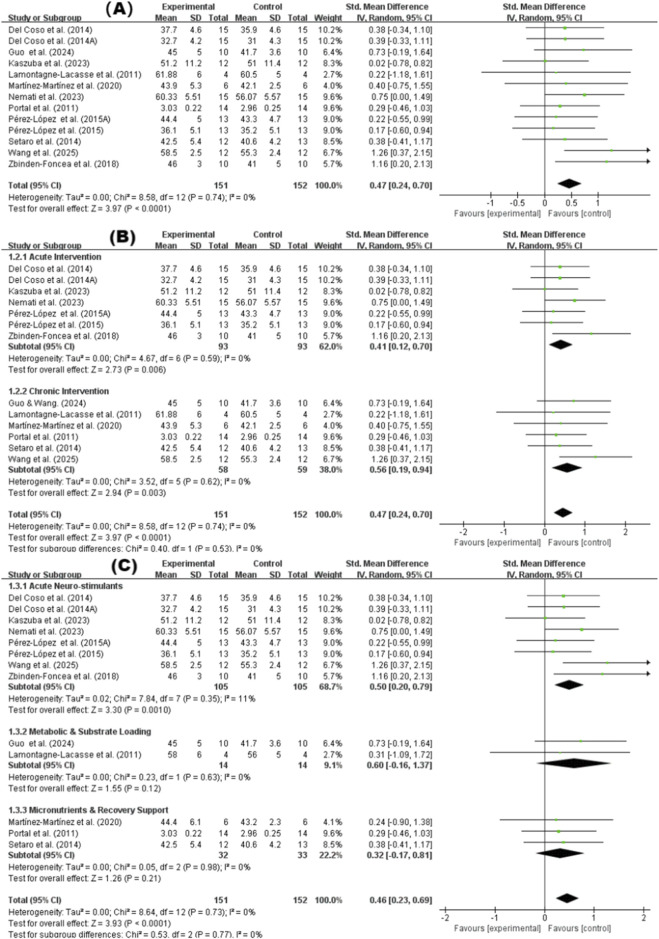
Vertical jump performance. **(A)** Overall effect; **(B)** Subgroup by intervention duration; **(C)** Subgroup by supplement type.

In the quantitative analysis of vertical jump performance, 11 studies were included (Del Coso et al., 2014; Guo and Wang, 2024; Kaszuba et al., 2023; Lamontagne-Lacasse et al., 2011; Martín-Martínez et al., 2020; Nemati et al., 2023; Pérez-López et al., 2015; Portal et al., 2011; Setaro et al., 2014; Wang et al., 2025; Zbinden-Foncea et al., 2018). The meta-analysis results revealed a significant difference (p < 0.0001) between supplementation and placebo trials concerning vertical jump performance (Figure 3A). The pooled standardized mean difference (SMD) was 0.47 (95% CI: 0.24, 0.70), with no observed heterogeneity (I^2^ = 0%). Subgroup analysis by intervention duration indicated that both acute (SMD = 0.41; p = 0.006) and chronic supplementation (SMD = 0.56; p = 0.003) significantly improved jumping performance (Figure 3B). Regarding supplement type, acute neuro-stimulants elicited significant improvements (SMD = 0.50; p = 0.001), whereas metabolic and substrate loading agents showed a non-significant trend toward improvement (SMD = 0.60; p = 0.12), and micronutrients and recovery support agents showed no significant effect (SMD = 0.32; p = 0.21) (Figure 3C). Although no statistical heterogeneity was detected (I^2^ ≈ 0%), this finding should be interpreted cautiously, as the number of included studies for some outcomes was limited, which may reduce the power to detect between-study heterogeneity.

#### 3.5.2 Muscle strength performance

**FIGURE 4:**
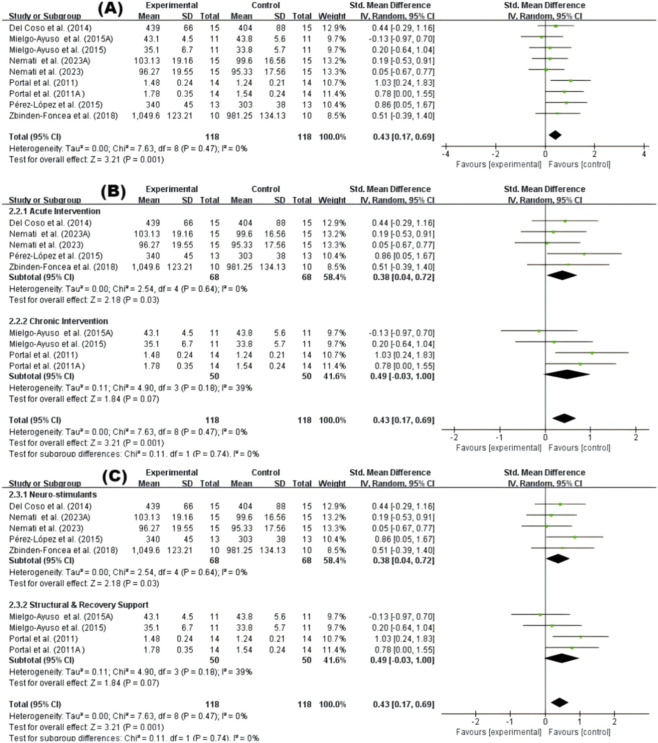
Muscle strength performance. **(A)** Overall effect **(B)** Subgroup by intervention duration **(C)** Subgroup by supplement type.

In the quantitative analysis of muscle strength, six studies contributed strength-related outcomes. When studies reported multiple strength measures, one prespecified representative outcome per study was prioritized for the primary analysis to reduce unit-of-analysis concerns, and alternative measures were explored in sensitivity analyses (Del Coso et al., 2014; Nemati et al., 2023; Pérez-López et al., 2015; Mielgo-Ayuso et al., 2015; Portal et al., 2011; Zbinden-Foncea et al., 2018). The meta-analysis results demonstrated a significant improvement in muscle strength following supplementation compared to placebo (SMD = 0.43; 95% CI: 0.17, 0.69; p = 0.001), with no observed heterogeneity (I^2^ = 0%) (Figure 4A). Subgroup analysis based on intervention duration revealed that both acute (SMD = 0.38; 95% CI: 0.04, 0.72; p = 0.03) and chronic interventions (SMD = 0.49; 95% CI: −0.03, 1.00; p = 0.07) showed a positive effect on strength performance, although the chronic subgroup did not reach statistical significance (Figure 4B). Similarly, subgroup analysis by supplement type indicated that caffeine significantly enhanced strength (SMD = 0.38; 95% CI: 0.04, 0.72; p = 0.03), while structural and recovery support agents showed a trend towards improvement (SMD = 0.49; 95% CI: −0.03, 1.00; p = 0.07) (Figure 4C).

#### 3.5.3 Agility performance

**FIGURE 5:**
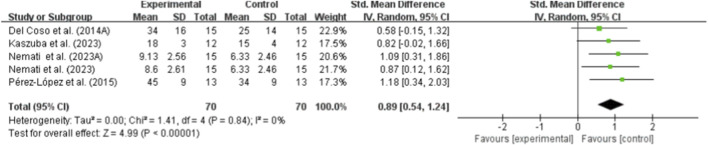
Agility and speed performance.

In the quantitative analysis of agility, four studies were included (Del Coso et al., 2014; Kaszuba et al., 2023; Nemati et al., 2023; Pérez-López et al., 2015). The results of the meta-analysis revealed a large and significant improvement in agility and speed performance following supplementation compared to placebo (SMD = 0.89; 95% CI: 0.54, 1.24; p < 0.00001). No heterogeneity was observed across the included studies (I^2^ = 0%, p = 0.84), indicating a high consistency in the ergogenic effects of the interventions on agility performance (Figure 5).

#### 3.5.4 Sport-specific technical skills

**FIGURE 6:**
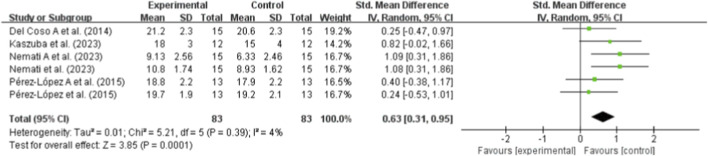
Sport-specific technical skills.

In the quantitative analysis of sport-specific technical skills, four studies were included (Del Coso et al., 2014; Kaszuba et al., 2023; Nemati et al., 2023; Pérez-López et al., 2015). These outcomes included spike-ball velocity (Del Coso et al., 2014; Pérez-López et al., 2015) and accuracy-/score-based attack and serve skill tests (Kaszuba et al., 2023; Nemati et al., 2023). The results of the meta-analysis demonstrated a significant improvement in technical skill performance following supplementation compared to placebo (SMD = 0.63; 95% CI: 0.31, 0.95; p < 0.001). The analysis showed negligible heterogeneity (I^2^ = 4%, p = 0.39), suggesting consistent positive effects of the interventions on the quality and accuracy of volleyball-specific actions (Figure 6).

### 3.6 Sensitivity analysis

Sensitivity analyses were performed by sequentially excluding individual studies from each meta-analysis. The direction and magnitude of the pooled effect sizes remained largely unchanged across all outcomes, and no substantial heterogeneity emerged. These findings indicate that the overall results were not driven by any single study and support the robustness of the primary analyses.

## 4 Discussion

The primary aim of this systematic review and meta-analysis was to evaluate the efficacy of nutritional supplementation strategies on physical performance and sport-specific skills in competitive volleyball players. To the best of our knowledge, this is the first quantitative synthesis to comprehensively examine these outcomes specifically within the volleyball cohort. The pooled analyses indicate that nutritional supplementation was associated with statistically significant improvements across the examined performance domains. With effects appearing more consistent for outcomes predominantly informed by acute caffeine-based trials. Specifically, supplementation demonstrated a large beneficial effect on agility and speed (SMD = 0.89) and sport-specific technical skills (SMD = 0.63), as well as a medium beneficial effect on vertical jump height (SMD = 0.47) and muscle strength (SMD = 0.43). These findings align with previous meta-analyses in other intermittent team sports, such as soccer and basketball, which have reported similar performance enhancements following ergogenic aid ingestion (Mielgo-Ayuso et al., 2019; Tan et al., 2022; Salinero et al., 2019). The findings indicate that certain supplementation strategies, particularly acute caffeine-based protocols, may serve as ergogenic aids for key volleyball-related attributes; however, evidence for non-caffeine supplements remains limited due to the small number of trials per supplement type and variation in protocols.

### 4.1 Practical implications for volleyball performance

While statistical significance confirms the efficacy of these interventions, the practical magnitude of these improvements is of greater relevance to coaches and athletes. The observed medium effect size for vertical jump (SMD = 0.47) translates to tangible performance gains. For instance, specific studies in our analysis reported absolute increases in jump height ranging from approximately 2.5–3.8 cm (Lamontagne-Lacasse et al., 2011; Setaro et al., 2014) or relative improvements of ∼5% (Del Coso et al., 2014). In the context of elite volleyball, anthropometric and biomechanical analyses indicate that jump height is a primary determinant of attack success and block effectiveness (Sattler et al., 2015). In elite volleyball, where performance margins are narrow, an increase of 3–4 cm in vertical jump height may be practically relevant for actions such as attacking and blocking. Similarly, the large effect size observed in agility (SMD = 0.89) suggests a substantial enhancement in on-court mobility. Volleyball rallies are characterized by short-duration, high-intensity multi-directional movements occurring within constrained spaces (Sheppard et al., 2007). The improved agility metrics observed in our analysis (Kaszuba et al., 2023; Nemati et al., 2023) directly correlate with an athlete’s ability to transition rapidly from a blocking action to a defensive dig, thereby increasing the team’s defensive coverage and transition efficiency.

Furthermore, these fractional enhancements must be interpreted within the paradigm of “marginal gains” in elite sport. Research indicates that the smallest worthwhile enhancement for competitive athletes is approximately 0.3%–1.0% of performance velocity or power (Hopkins et al., 1999). The improvements identified in our meta-analysis (e.g., ∼5% in jump height) far exceed this threshold, suggesting a profound impact on competitive outcomes. From a tactical perspective, the increased vertical displacement does not merely facilitate attack velocity; it fundamentally alters the geometry of the net exchange. A higher point of contact during blocking expands the “defensive shadow” cast into the opponent’s court, forcing attackers to attempt riskier trajectories or steeper angles, thereby increasing their unforced error rate (Silva et al., 2014). Additionally, considering that a volleyball player performs 60–100 maximal jumps per match (Mroczek et al., 2017), the cumulative effect of these supplement-induced gains is substantial. If nutritional strategies can mitigate the typical decrement in jump height observed in the fourth and fifth sets, they effectively preserve the team’s tactical integrity when match fatigue is at its peak.

### 4.2 Underlying physiological mechanisms

The comprehensive improvements observed across vertical jump, muscle strength, and agility can be mechanistically elucidated by examining the distinct physiological pathways targeted by the different supplement types. The robust effect sizes seen in agility (SMD = 0.89) and vertical jump (SMD = 0.47) are largely driven by the acute neuro-stimulant category. These explosive, motor-skill-dependent tasks benefit from the antagonism of adenosine *A*_*1*_ and *A*_*2A*_ receptors, which likely disinhibits the release of excitatory neurotransmitters such as dopamine and norepinephrine, thereby enhancing central nervous system (CNS) drive (Guest et al., 2021; Davis et al., 2003). This heightened CNS excitability may translate to increased motor unit recruitment and improved firing rates, offering a plausible explanation for the significant enhancements in rapid force development required for volleyball-specific actions (Kalmar and Cafarelli, 1999). Furthermore, at the peripheral level, caffeine has been suggested to sensitize ryanodine receptors, potentially facilitating sarcoplasmic reticulum Ca^2+^ release and optimizing excitation-contraction coupling even under fatigued conditions (Tarnopolsky, 2008; Zbinden-Foncea et al., 2018). These neural adaptations may plausibly contribute to the observed improvements in reactive agility, a finding that contrasts with some endurance-based studies where caffeine’s effect on neuromuscular precision was reported to be negligible (Pickering and Grgic, 2019).

In contrast, the gains in muscle strength and repeated high-intensity efforts appear to be supported by metabolic and recovery agents (Ismaeel, 2017; Mielgo-Ayuso et al., 2019). The efficacy of creatine observed in our analysis may be attributed to the expansion of the intramuscular phosphocreatine (PCr) pool, which optimizes the creatine kinase phosphagen shuttle (Wallimann et al., 1992). This mechanism is particularly relevant for volleyball, as it likely enhances the spatial and temporal buffering of adenosine triphosphate, thereby accelerating PCr resynthesis during the brief rest intervals between rallies (Kreider et al., 2017; Lamontagne-Lacasse et al., 2011). Regarding β-alanine, the observed benefits may be linked to augmented muscle carnosine content, which enhances intracellular buffering capacity. By mitigating the acidosis-induced inhibition of calcium sensitivity at the troponin C binding site (Dutka et al., 2012), β-alanine supplementation likely preserves contractile function during prolonged sets, a notion that is consistent with recent findings in other high-intensity intermittent sports (Sale et al., 2010; Guo and Wang, 2024; Trexler et al., 2015).

Finally, the role of structural support agents like HMB warrants specific attention. While the efficacy of HMB in general resistance-trained populations remains equivocal (Jakubowski et al., 2020; Durkalec-Michalski et al., 2017), our results suggest a potential benefit for volleyball athletes. This may be explained by the high volume of eccentric contractions (e.g., landing from jumps) inherent to volleyball, which induces significant muscle damage. HMB is thought to function via a dual signaling pathway: inhibiting the ubiquitin-proteasome proteolytic pathway to reduce protein breakdown and stimulating the mammalian target of rapamycin complex 1 (mTORC1) pathway to upregulate protein synthesis (Wilkinson et al., 2013; Wilson et al., 2013). Thus, in the specific context of competitive volleyball, HMB may act as a critical agent for mitigating exercise-induced muscle damage and facilitating recovery (Portal et al., 2011). In summary, our results suggest that the observed ergogenic effects are likely mediated by a synergistic combination of immediate neural potentiation and sustained metabolic and structural conditioning. It should be emphasized that the present meta-analysis does not directly test physiological mechanisms, and the proposed explanations are inferential and based on prior experimental evidence.

### 4.3 Effects on technical skills and cognitive function

An important finding of this meta-analysis is the observed association between supplementation and improvements in sport-specific technical skills (SMD = 0.63). While gross motor tasks (e.g., jumping) primarily rely on power output, volleyball skills such as serving, passing, and spiking are open-loop motor tasks that require high cognitive vigilance, decision-making, and fine motor control under time constraints (Fostinelli et al., 2019). The observed improvements in skill accuracy, particularly after acute caffeine ingestion (Kaszuba et al., 2023; Nemati et al., 2023), suggest that ergogenic benefits extend beyond muscular adaptations to include central cognitive processes. Research shows that caffeine can enhance vigilance and reduce the rating of perceived exertion by modulating central fatigue pathways (Doherty and Smith, 2005; Meeusen et al., 2013). This reduction in mental fatigue is especially important in volleyball, where technical errors accumulate in the later stages of a set due to cognitive lapses rather than physical exhaustion. By sustaining the “perceptual-motor” loop necessary for precise ball control, nutritional strategies can help mitigate the decline in technical proficiency, which may be relevant for maintaining technical performance in a sport where minimizing errors is critical (Pérez-López et al., 2015).

### 4.4 Limitations and future directions

Despite the promising findings, several limitations of this meta-analysis warrant consideration. First, most included trials were small, which limits statistical power and external validity and may lead to imprecise, and potentially inflated, effect estimates. In this context, low I^2^ values should not be interpreted as evidence of true between-study homogeneity because a limited number of studies reduces the ability to detect heterogeneity; therefore, the apparent consistency of effects should be interpreted with caution. Second, female athletes were underrepresented, accounting for only about 22% of the pooled sample. This restricts generalizability to female volleyball players and is particularly relevant because sex-specific physiological factors, including hormonal fluctuations across the menstrual cycle, may influence metabolic and neuromuscular responses to supplementation (Wickham and Spriet, 2019). Third, although all included studies were placebo-controlled, blinding integrity may have been compromised when supplements produced perceptible physiological effects. Such expectancy effects could have contributed to the magnitude of observed performance improvements (Saunders et al., 2017). Fourth, some trials reported multiple intervention arms or doses as well as multiple outcomes within the same performance domain. Although predefined procedures were applied to minimize disproportionate weighting, residual unit-of-analysis concerns cannot be fully excluded. Finally, supplementation protocols varied substantially in type, dose, duration, and timing, and performance-testing procedures also differed across studies. While subgroup analyses were conducted to explore acute versus chronic interventions and supplement categories, several subgroups were informed by few studies and should be viewed as exploratory and hypothesis-generating rather than definitive.

Future research should prioritize adequately powered trials with sex-stratified designs, and where feasible should report menstrual-cycle phase and hormonal contraceptive status to support sex-specific interpretation. Additionally, studies using ecologically valid match-play simulations are needed to determine whether laboratory-based improvements translate into meaningful competitive advantages across the demands of a full five-set match.

## 5 Conclusion

This systematic review and meta-analysis suggests that nutritional supplementation may be associated with improvements in several volleyball-relevant outcomes, with the most consistent evidence observed for agility and sport-specific technical skills. Notably, these effects were predominantly informed by acute caffeine-based trials, whereas evidence for non-caffeine supplements remains limited and heterogeneous. Small-to-moderate improvements were also observed in vertical jump performance and muscle strength, although conclusions regarding chronic metabolic or recovery-oriented agents are constrained by the small number of available studies. Collectively, these findings may inform evidence-based practice, particularly regarding acute caffeine strategies, while underscoring the need for larger, well-controlled, and ecologically valid trials to support supplement-specific recommendations beyond caffeine.

## Data Availability

The original contributions presented in the study are included in the article/Supplementary Material, further inquiries can be directed to the corresponding author.
